# (2*E*)-2-(4-Fluoro­benzyl­idene)hydrazinecarboxamide

**DOI:** 10.1107/S1600536811040797

**Published:** 2011-10-08

**Authors:** Hoong-Kun Fun, Tze Shyang Chia, Shridhar Malladi, Arun M. Isloor, Kammasandra N. Shivananda

**Affiliations:** a X-ray Crystallography Unit, School of Physics, Universiti Sains Malaysia, 11800 USM, Penang, Malaysia; b Medicinal Chemistry Section, Department of Chemistry, National Institute of Technology-Karnataka, Surathkal, Mangalore 575 025, India; c Schulich Faculty of Chemistry, Technion Israel Institute of Technology, Haifa 32000, Israel

## Abstract

In the title compound, C_8_H_8_FN_3_O, the semicarbazide group is close to being planar, with a maximum deviation of 0.020 (1) Å, and subtends a dihedral angle of 16.63 (9)° with its attached fluoro­benzene ring. In the crystal, mol­ecules are linked by N—H⋯O hydrogen bonds, forming layers lying parallel to the *bc* plane.

## Related literature

For background to semicarbazides and semicarbazones, see: Dogan *et al.* (1999 ▶); Pandeya & Dimmock (1993 ▶); Pandeya *et al.* (1998 ▶); Sriram *et al.* (2004 ▶); Yogeeswari *et al.* (2004 ▶); For further synthetic details, see: Furniss *et al.* (1978 ▶). For related structures, see: Fun *et al.* (2009*a* ▶,*b* ▶). For reference bond lengths, see: Allen *et al.* (1987 ▶).
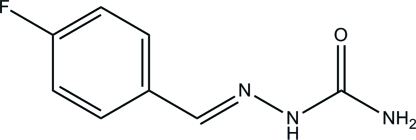


## Experimental

### 

#### Crystal data

C_8_H_8_FN_3_O
*M* *_r_* = 181.17
Monoclinic, 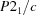

*a* = 16.522 (2) Å
*b* = 4.4381 (6) Å
*c* = 11.9457 (15) Å
β = 103.478 (3)°
*V* = 851.80 (19) Å^3^
*Z* = 4
Mo *K*α radiation
μ = 0.11 mm^−1^
*T* = 296 K
0.72 × 0.18 × 0.12 mm

#### Data collection

Bruker APEX DUO CCD diffractometer
Absorption correction: multi-scan (*SADABS*; Bruker, 2009 ▶) *T*_min_ = 0.923, *T*_max_ = 0.987
8746 measured reflections
2418 independent reflections
1657 reflections with *I* > 2σ(*I*)
*R*_int_ = 0.024

#### Refinement

*R*[*F* ^2^ > 2σ(*F* ^2^)] = 0.053
*wR*(*F* ^2^) = 0.209
*S* = 1.00
2418 reflections
130 parameters
H atoms treated by a mixture of independent and constrained refinement
Δρ_max_ = 0.31 e Å^−3^
Δρ_min_ = −0.21 e Å^−3^

### 

Data collection: *APEX2* (Bruker, 2009 ▶); cell refinement: *SAINT* (Bruker, 2009 ▶); data reduction: *SAINT*; program(s) used to solve structure: *SHELXTL* (Sheldrick, 2008 ▶); program(s) used to refine structure: *SHELXTL*; molecular graphics: *SHELXTL*; software used to prepare material for publication: *SHELXTL* and *PLATON* (Spek, 2009 ▶).

## Supplementary Material

Crystal structure: contains datablock(s) global, I. DOI: 10.1107/S1600536811040797/hb6436sup1.cif

Structure factors: contains datablock(s) I. DOI: 10.1107/S1600536811040797/hb6436Isup2.hkl

Supplementary material file. DOI: 10.1107/S1600536811040797/hb6436Isup3.cml

Additional supplementary materials:  crystallographic information; 3D view; checkCIF report

## Figures and Tables

**Table 1:** Hydrogen-bond geometry (Å, °)

*D*—H⋯*A*	*D*—H	H⋯*A*	*D*⋯*A*	*D*—H⋯*A*
N3—H1*N*3⋯O1^i^	0.88 (2)	2.07 (2)	2.8954 (19)	158 (2)
N2—H1*N*2⋯O1^ii^	0.92 (2)	2.00 (2)	2.9155 (19)	179 (2)

Symmetry codes: (i) 
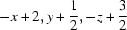
; (ii) 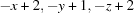.

**Fig. 1.**
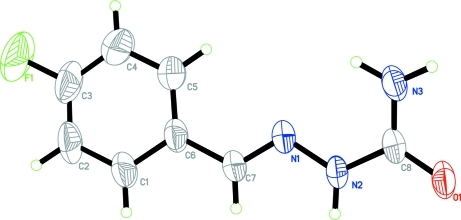
The molecular structure of the title compound with atom labels with 50% probability displacement ellipsoids.

**Fig. 2.**
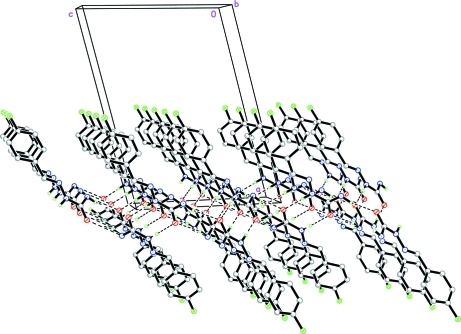
The crystal packing of the title compound. The dashed lines represent the hydrogen bonds.

## supplementary crystallographic information

### Comment

The semicarbazides, which are the raw material of semicarbazones, have been known to possess biological activities against many of the most common species of bacteria (Dogan *et al.*, 1999). Semicarbazones are of much interest due to their wide spectrum of antibacterial activities (Pandeya & Dimmock, 1993). Recently some workers have reviewed the bioactivity of semicarbazones and they have exhibited anticonvulsant (Pandeya *et al.*, 1998; Yogeeswari *et al.*, 2004) and antitubercular (Sriram *et al.*, 2004) properties. Accordingly and by considering the biological potential of semicarbazones, herein, we have synthesized the title compound to study its crystal structure.

The molecular structure of the title compound is shown in Fig. 1. The semicarbazone group (O1/N1–N3/C8) is essentially planar with maximum deviation of 0.020 (1) Å for atom N2. This plane makes dihedral angle of 16.63 (9)° with its terminal benzene ring (C1–C6). Bond lengths (Allen *et al.*, 1987) and angles are within normal ranges and are comparable to related structures (Fun *et al.*, 2009*a*,*b*).

In the crystal structure (Fig. 2), the molecules are interconnected by N3—H1N3···O1 and N2—H1N2···O1 hydrogen bonds (Table 1) forming two-dimensional networks parallel to *bc* plane.

### Experimental

Semicarbazide hydrochloride (0.86 g, 7.70 mmol) and freshly recrystallized sodium acetate (0.77 g, 9.40 mmol) were dissolved in water (10 ml) following a literature procedure (Furniss *et al.*, 1978). The reaction mixture was stirred at room temperature for 10 minutes. To this, 4-fluorobenzaldehyde (0.896 g, 7.23 mmol) was added and the mixture was shaken well. A little alcohol was added to dissolve the turbidity. The mixture was shaken for a further 10 minutes and allowed to stand. The title compound crystallizes out on standing for 6 h. The separated crystals were filtered, washed with cold water and recrystallized from ethanol to yield colourless needles. Yield: 0.98 g, 75.38%. *M.p.*: 506–508 K.

### Refinement

Atoms H1N2, H1N3 and H2N3 were located in a difference map and refined freely [N—H = 0.90 (2), 0.87 (2) and 0.91 (2) Å respectively]. The remaining H atoms were positioned geometrically [C—H = 0.93 Å] and refined using a riding model with *U*_iso_(H) = 1.2 *U*_eq_(C).

### Crystal data

**Table:**

C_8_H_8_FN_3_O	*F*(000) = 376
*M**_r_* = 181.17	*D*_x_ = 1.413 Mg m^−^^3^
Monoclinic, *P*2_1_/*c*	Mo *K*α radiation, λ = 0.71073 Å
Hall symbol: -P 2ybc	Cell parameters from 2666 reflections
*a* = 16.522 (2) Å	θ = 2.5–29.4°
*b* = 4.4381 (6) Å	µ = 0.11 mm^−^^1^
*c* = 11.9457 (15) Å	*T* = 296 K
β = 103.478 (3)°	Needle, colourless
*V* = 851.80 (19) Å^3^	0.72 × 0.18 × 0.12 mm
*Z* = 4	

### Data collection

**Table:**

Bruker APEX DUO CCD diffractometer	2418 independent reflections
Radiation source: fine-focus sealed tube	1657 reflections with *I* > 2σ(*I*)
graphite	*R*_int_ = 0.024
φ and ω scans	θ_max_ = 29.9°, θ_min_ = 1.3°
Absorption correction: multi-scan (*SADABS*; Bruker, 2009)	*h* = −23→23
*T*_min_ = 0.923, *T*_max_ = 0.987	*k* = −6→6
8746 measured reflections	*l* = −16→15

### Refinement

**Table:**

Refinement on *F*^2^	Primary atom site location: structure-invariant direct methods
Least-squares matrix: full	Secondary atom site location: difference Fourier map
*R*[*F*^2^ > 2σ(*F*^2^)] = 0.053	Hydrogen site location: inferred from neighbouring sites
*wR*(*F*^2^) = 0.209	H atoms treated by a mixture of independent and constrained refinement
*S* = 1.00	*w* = 1/[σ^2^(*F*_o_^2^) + (0.1446*P*)^2^ + 0.0418*P*] where *P* = (*F*_o_^2^ + 2*F*_c_^2^)/3
2418 reflections	(Δ/σ)_max_ < 0.001
130 parameters	Δρ_max_ = 0.31 e Å^−^^3^
0 restraints	Δρ_min_ = −0.21 e Å^−^^3^

### Special details

**Table:**

Geometry. All e.s.d.'s (except the e.s.d. in the dihedral angle between two l.s. planes) are estimated using the full covariance matrix. The cell e.s.d.'s are taken into account individually in the estimation of e.s.d.'s in distances, angles and torsion angles; correlations between e.s.d.'s in cell parameters are only used when they are defined by crystal symmetry. An approximate (isotropic) treatment of cell e.s.d.'s is used for estimating e.s.d.'s involving l.s. planes.
Refinement. Refinement of *F*^2^ against ALL reflections. The weighted *R*-factor *wR* and goodness of fit *S* are based on *F*^2^, conventional *R*-factors *R* are based on *F*, with *F* set to zero for negative *F*^2^. The threshold expression of *F*^2^ > σ(*F*^2^) is used only for calculating *R*-factors(gt) *etc*. and is not relevant to the choice of reflections for refinement. *R*-factors based on *F*^2^ are statistically about twice as large as those based on *F*, and *R*- factors based on ALL data will be even larger.

### Fractional atomic coordinates and isotropic or equivalent isotropic displacement parameters (Å^2^)

**Table:**

	*x*	*y*	*z*	*U*_iso_*/*U*_eq_	
F1	0.51936 (9)	1.2636 (4)	1.15848 (18)	0.1170 (6)	
O1	1.01013 (7)	0.7059 (3)	0.87156 (9)	0.0508 (3)	
N1	0.83497 (7)	0.8821 (3)	0.97516 (10)	0.0448 (3)	
N2	0.90494 (8)	0.7354 (3)	0.96093 (11)	0.0470 (3)	
N3	0.91237 (8)	1.0690 (3)	0.81619 (11)	0.0493 (4)	
C1	0.71134 (11)	0.8578 (5)	1.18840 (15)	0.0637 (5)	
H1A	0.7461	0.7333	1.2413	0.076*	
C2	0.63885 (11)	0.9701 (6)	1.21288 (16)	0.0705 (5)	
H2A	0.6244	0.9217	1.2814	0.085*	
C3	0.58977 (12)	1.1518 (5)	1.1342 (2)	0.0740 (6)	
C4	0.60851 (13)	1.2285 (6)	1.0325 (2)	0.0855 (7)	
H4A	0.5735	1.3543	0.9805	0.103*	
C5	0.68021 (11)	1.1161 (5)	1.00849 (17)	0.0669 (5)	
H5A	0.6937	1.1662	0.9394	0.080*	
C6	0.73235 (9)	0.9300 (4)	1.08559 (13)	0.0493 (4)	
C7	0.80796 (9)	0.8014 (4)	1.06141 (13)	0.0504 (4)	
H7A	0.8371	0.6560	1.1109	0.060*	
C8	0.94581 (8)	0.8362 (3)	0.88136 (11)	0.0405 (3)	
H1N3	0.9423 (12)	1.145 (5)	0.7725 (18)	0.064 (5)*	
H1N2	0.9306 (12)	0.599 (5)	1.0133 (16)	0.061 (5)*	
H2N3	0.8717 (14)	1.181 (5)	0.8357 (19)	0.074 (6)*	

### Atomic displacement parameters (Å^2^)

**Table:**

	*U*^11^	*U*^22^	*U*^33^	*U*^12^	*U*^13^	*U*^23^
F1	0.0854 (10)	0.1393 (14)	0.1524 (15)	0.0374 (9)	0.0807 (10)	0.0080 (10)
O1	0.0544 (6)	0.0586 (7)	0.0499 (6)	0.0023 (4)	0.0334 (5)	−0.0022 (4)
N1	0.0447 (6)	0.0544 (7)	0.0417 (6)	0.0019 (5)	0.0228 (5)	−0.0006 (5)
N2	0.0490 (7)	0.0566 (7)	0.0446 (7)	0.0073 (5)	0.0296 (5)	0.0048 (5)
N3	0.0569 (7)	0.0541 (7)	0.0457 (7)	−0.0004 (5)	0.0299 (6)	0.0032 (5)
C1	0.0563 (9)	0.0936 (13)	0.0502 (9)	0.0081 (8)	0.0304 (7)	0.0076 (8)
C2	0.0662 (10)	0.0974 (15)	0.0620 (10)	−0.0021 (9)	0.0438 (9)	−0.0068 (10)
C3	0.0558 (10)	0.0863 (14)	0.0938 (15)	0.0098 (8)	0.0457 (10)	−0.0057 (11)
C4	0.0695 (12)	0.1019 (16)	0.0965 (17)	0.0321 (11)	0.0423 (12)	0.0232 (13)
C5	0.0631 (10)	0.0820 (12)	0.0656 (11)	0.0196 (8)	0.0353 (8)	0.0179 (9)
C6	0.0457 (7)	0.0643 (9)	0.0448 (7)	0.0019 (6)	0.0244 (6)	−0.0001 (6)
C7	0.0482 (8)	0.0673 (9)	0.0425 (8)	0.0090 (6)	0.0245 (6)	0.0085 (6)
C8	0.0461 (7)	0.0452 (7)	0.0361 (6)	−0.0067 (5)	0.0215 (5)	−0.0085 (5)

### Geometric parameters (Å, °)

**Table:**

F1—C3	1.3568 (19)	C1—H1A	0.9300
O1—C8	1.2393 (16)	C2—C3	1.355 (3)
N1—C7	1.2661 (18)	C2—H2A	0.9300
N1—N2	1.3714 (16)	C3—C4	1.365 (3)
N2—C8	1.3634 (17)	C4—C5	1.376 (2)
N2—H1N2	0.90 (2)	C4—H4A	0.9300
N3—C8	1.3333 (18)	C5—C6	1.379 (2)
N3—H1N3	0.87 (2)	C5—H5A	0.9300
N3—H2N3	0.91 (2)	C6—C7	1.4621 (19)
C1—C2	1.390 (2)	C7—H7A	0.9300
C1—C6	1.389 (2)		
			
C7—N1—N2	115.71 (12)	C3—C4—C5	118.7 (2)
C8—N2—N1	119.96 (12)	C3—C4—H4A	120.6
C8—N2—H1N2	118.4 (12)	C5—C4—H4A	120.6
N1—N2—H1N2	120.3 (12)	C4—C5—C6	120.79 (17)
C8—N3—H1N3	115.9 (13)	C4—C5—H5A	119.6
C8—N3—H2N3	120.3 (14)	C6—C5—H5A	119.6
H1N3—N3—H2N3	120 (2)	C5—C6—C1	118.85 (14)
C2—C1—C6	120.56 (17)	C5—C6—C7	122.08 (14)
C2—C1—H1A	119.7	C1—C6—C7	119.06 (15)
C6—C1—H1A	119.7	N1—C7—C6	121.99 (14)
C3—C2—C1	118.24 (16)	N1—C7—H7A	119.0
C3—C2—H2A	120.9	C6—C7—H7A	119.0
C1—C2—H2A	120.9	O1—C8—N3	123.66 (12)
F1—C3—C2	118.27 (19)	O1—C8—N2	119.18 (13)
F1—C3—C4	118.9 (2)	N3—C8—N2	117.15 (12)
C2—C3—C4	122.86 (16)		
			
C7—N1—N2—C8	−170.19 (13)	C4—C5—C6—C7	178.57 (19)
C6—C1—C2—C3	−0.3 (3)	C2—C1—C6—C5	0.3 (3)
C1—C2—C3—F1	−179.4 (2)	C2—C1—C6—C7	−178.40 (17)
C1—C2—C3—C4	0.0 (4)	N2—N1—C7—C6	−177.87 (13)
F1—C3—C4—C5	179.6 (2)	C5—C6—C7—N1	8.7 (3)
C2—C3—C4—C5	0.2 (4)	C1—C6—C7—N1	−172.65 (16)
C3—C4—C5—C6	−0.1 (4)	N1—N2—C8—O1	178.19 (12)
C4—C5—C6—C1	−0.1 (3)	N1—N2—C8—N3	−3.3 (2)

### Hydrogen-bond geometry (Å, °)

**Table:**

*D*—H···*A*	*D*—H	H···*A*	*D*···*A*	*D*—H···*A*
N3—H1N3···O1^i^	0.88 (2)	2.07 (2)	2.8954 (19)	158 (2)
N2—H1N2···O1^ii^	0.92 (2)	2.00 (2)	2.9155 (19)	179 (2)

Symmetry codes: (i) −*x*+2, *y*+1/2, −*z*+3/2; (ii) −*x*+2, −*y*+1, −*z*+2.

## References

[bb1] Allen, F. H., Kennard, O., Watson, D. G., Brammer, L., Orpen, A. G. & Taylor, R. (1987). *J. Chem. Soc. Perkin Trans. 2*, pp. S1–19.

[bb2] Bruker (2009). *SADABS*, *APEX2* and *SAINT* Bruker AXS Inc., Madison, Wisconsin, USA.

[bb3] Dogan, H. N., Duran, A. & Yemni, E. (1999). *Drug Metab. Drug Interact.* **15**, 187–195. 10.1515/dmdi.1999.15.2-3.187 10707124

[bb4] Fun, H.-K., Goh, J. H., Padaki, M., Malladi, S. & Isloor, A. M. (2009*a*). *Acta Cryst.* E**65**, o1591–o1592. 10.1107/S1600536809022284 PMC2969411 21582866

[bb5] Fun, H.-K., Yeap, C. S., Padaki, M., Malladi, S. & Isloor, A. M. (2009*b*). *Acta Cryst.* E**65**, o1619–o1620. 10.1107/S1600536809022521 PMC2969237 21582889

[bb6] Furniss, B. S., Hannaford, A. J., Rogers, V., Smith, P. W. G. & Tatchell, A. R. (1978). *Vogel’s Textbook of Practical Organic Chemistry*, 4th ed., p. 1112. London: ELBS.

[bb7] Pandeya, S. N. & Dimmock, J. R. (1993). *Pharmazie*, **48**, 659–666. 8234398

[bb8] Pandeya, S. N., Misra, V., Singh, P. N. & Rupainwar, D. C. (1998). *Pharmacology*, **37**, 17–22. 10.1006/phrs.1997.0250 9503475

[bb9] Sheldrick, G. M. (2008). *Acta Cryst.* A**64**, 112–122. 10.1107/S0108767307043930 18156677

[bb10] Spek, A. L. (2009). *Acta Cryst.* D**65**, 148–155. 10.1107/S090744490804362X PMC2631630 19171970

[bb11] Sriram, D., Yogeeswari, P. & Thirumurugan, R. S. (2004). *Bioorg. Med. Chem. Lett.* **14**, 3923–3924. 10.1016/j.bmcl.2004.05.060 15225698

[bb12] Yogeeswari, P., Sriram, D., Pandeya, S. N. & Stables, J. P. (2004). *Farmaco*, **59**, 609–613. 10.1016/j.farmac.2004.01.009 15262530

